# A Novel Fracture Prediction Model Using Machine Learning in a Community‐Based Cohort

**DOI:** 10.1002/jbm4.10337

**Published:** 2020-02-10

**Authors:** Sung Hye Kong, Daehwan Ahn, Buomsoo (Raymond) Kim, Karthik Srinivasan, Sudha Ram, Hana Kim, A Ram Hong, Jung Hee Kim, Nam H Cho, Chan Soo Shin

**Affiliations:** ^1^ Department of Internal Medicine Seoul National University College of Medicine Seoul Republic of Korea; ^2^ Department of Operations, Information and Decisions, Wharton School University of Pennsylvania Philadelphia PA USA; ^3^ Department of Management Information Systems, Eller College of Management University of Arizona Tucson AZ USA; ^4^ Department of Internal Medicine Chonnam National University Hwasun Hospital Chonnam; ^5^ Department of Preventive Medicine Ajou University School of Medicine Suwon Republic of Korea

**Keywords:** FRACTURE, MACHINE LEARNING, PREDICTION MODEL, PROSPECTIVE COHORT

## Abstract

The prediction of fracture risk in osteoporotic patients has been a topic of interest for decades, and models have been developed for the accurate prediction of fracture, including the fracture risk assessment tool (FRAX). As machine‐learning methodologies have recently emerged as a potential model for medical prediction tools, we aimed to develop a novel fracture prediction model using machine‐learning methods in a prospective community‐based cohort. In this study, 2227 participants (1257 females) with a baseline bone mineral density (BMD) and trabecular bone score were enrolled from the Ansung cohort. The primary endpoint was the fragility fractures reported by patients or confirmed by X‐rays. We used 3 different models: CatBoost, support vector machine (SVM), and logistic regression. During a mean 7.5‐year follow‐up (range, 2.5 to 10 years), fragility fractures occurred in 537 (25.6%) of participants. In predicting total fragility fractures, the area under the curve (AUC) values of the CatBoost, SVM, and logistic regression models were 0.688, 0.500, and 0.614, respectively. The AUC value of CatBoost was significantly better than that of FRAX (0.663; *p* < 0.001), whereas the the SVM and logistic regression models were not. Compared with the conventional models such as SVM and logistic regression, the CatBoost model had the best performance in predicting total fragility fractures (*p* < 0.001). According to feature importance in the CatBoost model, the top predicting factors (listed in order) were total hip, lumbar spine, and femur neck BMD, subjective arthralgia score, serum creatinine, and homocysteine. The latter three factors were listed higher than conventional predictors such as age or previous fracture history. In summary, we hereby report the development of a prediction model for fragility fractures using a machine‐learning method, CatBoost, which outperforms the FRAX model as well as two conventional machine‐learning models. The model was also able to propose novel high‐ranking predictors. © 2020 The Authors. *JBMR Plus* published by Wiley Periodicals, Inc. on behalf of American Society for Bone and Mineral Research.

## Introduction

Fragility fracture has become a major socioeconomic issue in an aging society. The incidence of osteoporosis has been reported to be 12.9% in men and 24.0% in women over 50 years of age, and the frequency of osteoporotic fractures is continuously increasing by an annual average of 15.2% in Korea.^1^ Fragility fracture and its socioeconomic costs also increase along with the incidence of osteoporosis,^1^ which makes the prediction and prevention of particular importance currently.

Although bone mineral density (BMD) is a good predictor of fracture risk, many fractures occur in patients with osteopenia.^2^ To improve fracture prediction, the fracture risk assessment tool (FRAX; The University of Sheffield, Sheffield, UK) was developed as a fracture risk assessment tool using clinical factors in addition to BMD.^3^ As FRAX is an excellent prediction tool, it is increasingly used to guide treatment decisions, and has been integrated into many clinical practice guidelines.^4^

Recently, machine‐learning methodologies have emerged in medical prediction models, especially in cardiovascular disease.^5^, ^6^ In a similar way, this new approach might improve the performance of current fracture prediction models by including all possible variables such as the BMD of all sites as well as trabecular bone score (TBS) data. Also, the new model could suggest novel factors that could influence the fracture by calculating all variables through a deep learning network. Although there are a few studies in osteoporosis and fracture prediction using machine learning,^7^, ^8^, ^9^ a fracture‐prediction machine‐learning model with a longitudinal, large‐sized cohort study including BMD and TBS has not been developed.

There are various machine‐learning techniques such as support vector machine (SVM), and gradient boosting models like XGboost and CatBoost (for “categorical boosting”). Gradient boosting is a powerful machine‐learning technique typically used in developing decision trees, which could be done without extensive data training like other machine‐learning techniques. Among the gradient boosting techniques, CatBoost is the most recently developed model with excellent performance, which can handle categorical features without preprocessing to lower the chances of overfitting to make more generalized models.^10^

In our study, we aimed to develop a prediction model of fragility fractures and discover novel risk factors using a machine‐learning method in a large‐sized longitudinal community‐based cohort study.

## Materials and Methods

### Study population

This study was based on data obtained from the Ansung cohort study, which is an ongoing prospective study that began in 2001 and is supported by the National Genome Research Institute of the Korea Centers for Disease Control and Prevention (Cheongju, Korea). The study is part of the Korean Genome Epidemiology Study (KoGES), a large community‐based epidemiological survey that consists of a population‐based sample of Korean men and women aged 40 to 69 years old. Participants were residents of Ansung who had lived in the survey area for at least 6 months before enrollment. Detailed information on the selection criteria and sampling plan for the cohort study has been published previously.^11^, ^12^ The study protocol was approved by the Korea Centers for Disease Control and Prevention Institutional Review Board. The study was carried out following the World Medical Association Declaration of Helsinki — Ethical Principles for Medical Research. Consent was obtained from each patient after a full explanation of the purpose and nature of all procedures.

A total of 5018 participants completed a baseline examination in 2001 and were surveyed biennially. The BMD measurement began at the fourth wave (2007 to 2008). At the time of the fourth wave of the cohort, 3224 participants remained in the survey. For this analysis, we excluded 997 participants whose dual‐energy X‐ray absorptiometry (DXA) data were unavailable at the fourth wave. For the final analysis, 2227 participants were eligible.

### Fragility fractures

Fragility fractures were defined as fractures that resulted from no identifiable trauma or a minimal trauma such as a fall from a standing height or less, which included both the self‐report by patients and morphometric fractures confirmed by X‐rays. For the patient‐reported clinical fractures, face‐to‐face or telephone interviews were used to inquire about fractures. For the morphometric fracture confirmed by X‐rays, anterior, middle, and posterior vertebral heights were measured using the method described by Eastell and colleagues.^13^ Anterior to posterior, middle to posterior, and posterior to posterior above and below ratios were calculated. The vertebral fracture was defined if any of the abovementioned ratios were more than 3 standard deviations (SDs) below the normal mean for the vertebral level, as described in our previous report.^14^

### Health questionnaires and measurements parameters

Interviews obtained data on lifestyle and sociodemographic factors including age, sex, previous medical history, drinking and smoking status, physical activity, and menopausal age and status at the baseline. Participants with diabetes were defined as those who answered to have diabetes or those who have reached the thresholds for fasting plasma glucose ≥126 mg/dL or HbA1c ≥6.5%.^15^ Ever smokers were defined as those who had smoked >five packs of cigarettes during their lifetime. Usual drinkers were defined as those who consumed >5 g of ethanol/day.

Physical activity (PA) was determined by asking participants how often they exercised each week using the Korean version of the International Physical Activity Questionnaire (IPAQ). Based on the Ainsworth and colleagues’ compendium,^16^ an average metabolic equivalent (MET) score was derived for each type of activity. The following values were then used for the analysis of IPAQ data: walking, 3.3 METs; moderate PA, 4.0 METs; vigorous PA, 8.0 METs. A total PA (MET‐hours/week) was defined as the sum of the weekly METs for walking, moderate PA, and vigorous PA.

The arthralgia score was screened for any subjective arthritic pain with the 0 to 10 numeric rating scale, by which participants rate their current pain intensity from 0 (“no pain”) to 10 (“worst possible pain”) at the time of the interview. Height and body weight were measured based on standard methods by trained staff using a scale and a wall‐mounted extensometer with participants wearing lightweight clothes. BMI was calculated as the weight divided by height squared (kg/m^2^).

Cognitive impairment was evaluated using the Korean mini‐mental status examination (K‐MMSE), which is a 30‐item questionnaire specifically developed and validated for assessing the general cognitive function of older Korean individuals.^17^ The results are scored from 0 to 30 points, with scores of ≥23 points indicating normal cognition, scores of 17 to 22 points indicating mild cognitive impairment, and scores of <17 points indicating moderate‐to‐severe impairment. Depressive symptoms were assessed using the 15‐item Korean geriatric depression scale (K‐GDS).^18^ The results are scored from 0 to 15 points, with scores of >10 points considered suggestive of depressive mood.

### Laboratory assessments

At the baseline, the blood samples were acquired in the morning fasting status (14 hours of fasting for all participants). Plasma was separated immediately by centrifuge (2000 rpm, 20 min, at 4°C), and measurements were conducted immediately. Plasma glucose level was measured using the hexokinase method (ADVIA 1650 Auto Analyzer; Bayer, Leverkusen, Germany), and the plasma insulin level was measured using the IRMA test kit (BioSource Europe SA, Nivelles, Belgium). Fasting total cholesterol, high‐density lipoprotein cholesterol (HDL‐C), low‐density lipoprotein cholesterol (LDL‐C), and triglyceride (TG) levels were measured enzymatically using the Hitachi 747 chemistry analyzer (Hitachi, Tokyo, Japan). The HbA1c level was determined using high‐performance liquid chromatography by the Bio‐Rad Variant II HbA1c analyzer (Bio‐Rad, Montreal, Quebec, Canada). Homeostatic model assessment of insulin resistance (HOMA‐IR) was computed using the following formula:

$$\text{HOMA}-\mathrm{IR}=\text{fasting plasma insulin}\;\left(\mathrm{\mu IU}/\mathrm{mL}\right)\times \text{fasting plasma glucose}\;\left(\mathrm{mg}/\mathrm{dL}\right)\times 0.0555/22.5.$$

### Measurements of BMD, TBS, and calculations of FRAX

The BMD (grams/cm^2^) of skeletal sites (lumbar spine, femoral neck, and total hip) and muscle mass were measured using DXA (GE Prodigy; GE Healthcare, Chicago, IL, USA) and analyzed (enCORE Software version 11.0; GE Healthcare) according to the manufacturer's guidelines at baseline. The BMD precision error (% of the coefficient of variation [CV]) was 1.7% for the lumbar spine, 1.8% for the femoral neck, and 1.7% for the total hip. For the lumbar spine BMD, the L1 to L4 value was chosen for analysis. When L1 to L4 was not suitable for analysis because of a compression fracture or severe sclerotic change, L2 to L4 was used. All TBS measurements were retrospectively performed using TBS iNsight software, version 2.0.0.1 (Medimaps Group SA, Geneva, Switzerland) utilizing spine DXA files from the database to ensure that all investigators were blinded to all clinical parameters and outcomes. The software used the raw DXA image of the anteroposterior spine for the same region of interest as the BMD measurement. Instruments were calibrated using anthropomorphic phantoms.

The World Health Organization's 10‐year absolute risks of hip and osteoporotic fracture (FRAX scores) were calculated using the University of Sheffield online Korea‐specific FRAX tool (https://www.sheffield.ac.uk/FRAX/tool.aspx?country=25). The FRAX algorithm includes the following parameters: femoral neck BMD *T*‐score, lumbar TBS score, age, sex, BMI, previous history of fracture, parental history of hip fracture, secondary osteoporosis, current smoking status, recent use of corticosteroids, presence of rheumatoid arthritis, and ≥ three alcoholic beverages per day.

### Machine‐learning techniques used

We implemented a new gradient‐boosting algorithm, CatBoost, which successfully manages categorical features and outperforms existing state‐of‐the‐art machine‐learning algorithms on popular publicly available data sets.^10^ When developing the algorithm, we passed on the indices of categorical features to the function. By doing so, the algorithm can discriminate between categorical variables and continuous variables, supporting more reliable and efficient training. The latest versions in September, 2019 of the CatBoost package (https://github.com/catboost/catboost) and Python programming language were utilized for implementation.

To further evaluate the performance of the CatBoost model, the logistic regression and SVM models were tested in comparison. Logistic regression is a widely used model in a variety of fields, including medical research. SVM is a machine‐learning algorithm that is preferred in many studies because of its ease of use, high prediction accuracy, and robustness to overfitting.^19^ Both the SVM and logistic regression models come with the Python programming language, along with the latest version of the Scikit‐learn package (https://scikit-learn.org/stable/). All clinical variables from the cohort have been included for building both conventional and machine‐learning models.

SHAP (Shapley additive explanation) values were used to evaluate feature importance (https://github.com/slundberg/shap).^20^, ^21^ The SHAP value measures how much each feature in the model contributes, either positively or negatively, similar to coefficient values in logistic regression.

### Performance evaluation

We assessed and evaluated the performance of the prediction models in terms of the area under the curve (AUC) score calculated by randomly selected threefold cross‐validation for 1000 times. The AUC measures the performance of a classifier in terms of its ability to classify positive instances correctly.^22^ K‐fold validation is a model validation technique that prevents the overfitting of predictive models to training data.^23^ In this method, the original training data set is randomly split into a quantity of k equal‐sized exclusive subsets; of the k subsets, a single subsample is retained as the validation data, and the remaining k − 1 subsets are used as training data in each iteration, followed by averaging the model performance results.

**Figure 1:**
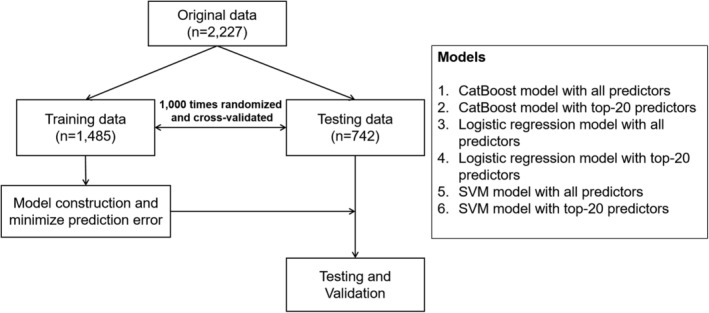
Study participants and used models. SVM = support vector machine.

An iterated threefold cross‐validation was performed (*n* = 1000) for each method (ie, CatBoost, logistic regression, SVM; each model with all variables or top‐20 variables) to obtain a robust AUC score for each method and the corresponding 95% CI using the standard error generated through the training/test data sampled without replacement. Table 3 shows the comparison of the robust AUC scores of each method with the AUC of FRAX scores computed over all of the patients in the cohort (Fig. 1).

### Statistical analysis

In baseline characteristics, depending on the distribution, continuous parameters are presented as means with SDs, and categorical data are presented as proportions. Comparisons between groups were analyzed by performing Student's *t* test, whereas a χ^2^ test was used for categorical variables. A *p* value <0.05 was considered significant. Statistical analyses were performed using the SPSS 24.0 statistical package (IBM, Armonk, NY, USA) and R software (R Foundation for Statistical Computing, Vienna, Austria; http://www.r-project.org).

## Results

### Clinical characteristics

There were 2227 participants included in the analysis. The mean follow‐up duration was 7.5 years (range, 2 to 10 years). The average age was 61.2 ± 8.7 years old, 1257 (56.4%) of participants were female, which was the more prevalent sex in patients with fractures (*p* = 0.008). Patients with fractures also had later menarche (*p* < 0.001), experienced more previous fractures (*p* < 0.001), and were more commonly diagnosed with osteoporosis and osteoarthritis (*p* < 0.001 and *p* < 0.003, respectively) than those without fractures. Moreover, patients with fractures had higher arthralgic pain scores, lower cognitive function scores, and higher geriatric depression scores than those without fractures. BMD for lumbar, femur neck, and total hip, and lumbar TBS score were significantly lower in those with fractures than in those without (0.956 ± 0.192, 1.030 ± 0.184 g/cm^2^ for lumbar BMD; 0.793 ± 0.139, 0.858 ± 0.142 g/cm^2^ in femur neck BMD; 0.850 ± 0.148, 0.924 ± 0.151 g/cm^2^ in total hip BMD; 1.357 ± 0.097, 1.392 ± 0.094 in TBS, respectively, all *p* < 0.001). FRAX scores with and without BMD and FRAX score with TBS score were all higher in those with fractures than in those without fractures (FRAX score with TBS for major fracture 5.5 ± 3.6, 4.0 ± 2.6%; FRAX score with TBS for hip fracture 1.5 ± 1.9, 0.8 ± 1.2%, respectively, *p* < 0.001; Table 1).

**Table 1:** Clinical Characteristics of Participants

	Total (*n* = 2227)	Without fracture (*n* = 1690)	With fracture (*n* = 537)	*p*
Age, years	61.2 ± 8.7	60.4 ± 8.7	63.7 ± 8.2	<0.001
Female	1257 (56.4)	927 (54.9%)	330 (61.5%)	0.008
BMI, kg/m^2^	24.4 ± 3.3	24.4 ± 3.2	24.3 ± 3.3	0.551
Menarche, years	16.1 ± 1.9	16.0 ± 1.8	16.4 ± 1.9	<0.001
Menopause, years	46.5 ± 10.7	46.0 ± 10.9	47.8 ± 10.0	0.203
Ever smoker	746 (33.6%)	580 (34.4%)	166 (31.0%)	0.166
Ever drinker	349 (16.9%)	266 (17.1%)	83 (16.0%)	0.603
History of previous fracture	206 (9.3%)	120 (7.1%)	86 (16.0%)	<0.001
Diabetes	284 (12.8%)	220 (13.0%)	64 (12.0%)	0.564
Hypertension	934 (1.8%)	593 (35.2%)	210 (39.3%)	0.188
Osteoporosis	514 (23.1%)	338 (20.0%)	176 (32.9%)	<0.001
Arthritis	866 (39.8%)	968 (58.4%)	341 (66.0%)	0.003
Arthralgia, score	1.6 ± 3.1	1.4 ± 2.1	2.1 ± 5.0	0.001
K‐MMSE, score	23.2 ± 6.2	23.6 ± 6.0	22.2 ± 6.5	0.001
K‐GDS, score	4.3 ± 4.0	4.0 ± 3.9	5.1 ± 4.2	<0.001
Hba1c, %	5.9 ± 1.0	5.9 ± 1.0	5.8 ± 1.0	0.774
Creatinine, mg/dL	0.9 ± 0.2	0.9 ± 0.2	0.9 ± 0.2	0.007
ALT, mg/dL	24.9 ± 16.6	25.0 ± 16.7	24.7 ± 16.3	0.652
AST, mg/dL	27.2 ± 13.3	27.1 ± 12.6	27.7 ± 15.1	0.348
CRP, mg/dL	1.8 ± 5.2	1.7 ± 5.0	1.9 ± 5.7	0.510
Homocysteine, μmol/L	12.1 ± 5.0	12.1 ± 5.1	12.2 ± 4.6	0.579
TSH, μIU/mL	1.7 ± 1.7	1.7 ± 1.8	1.6 ± 1.4	0.146
HOMA‐β cell	106.0 ± 82.6	105.4 ± 87.8	107.8 ± 63.6	0.496
Lumbar BMD, g/cm^2^	1.007 ± 0.194	1.030 ± 0.184	0.956 ± 0.192	<0.001
Femur neck BMD, g/cm^2^	0.834 ± 0.146	0.858 ± 0.142	0.793 ± 0.139	<0.001
Total hip BMD, g/cm^2^	0.899 ± 0.154	0.924 ± 0.151	0.850 ± 0.148	<0.001
Lumbar TBS, score	1.406 ± 0.112	1.392 ± 0.094	1.357 ± 0.097	<0.001
Follow‐up duration, years	7.5 ± 1.6	7.7 ± 1.3	6.9 ± 2.3	<0.001
Mortality	128 (5.7%)	105 (6.2%)	23 (4.3%)	0.117
FRAX (major, without BMD), %	5.2 ± 3.1	4.9 ± 2.8	6.1 ± 3.6	<0.001
FRAX (hip, without BMD), %	1.6 ± 1.6	1.4 ± 1.5	2.0 ± 1.8	<0.001
FRAX (major, with BMD), %	4.5 ± 2.9	4.2 ± 2.7	5.5 ± 3.4	<0.001
FRAX (hip, with BMD), %	1.1 ± 1.7	0.9 ± 1.5	1.5 ± 2.0	<0.001
FRAX (major, with TBS), %	4.4 ± 2.9	4.0 ± 2.6	5.5 ± 3.6	<0.001
FRAX (hip, with TBS), %	0.9 ± 1.5	0.8 ± 1.2	1.5 ± 1.9	<0.001

Continuous variables are expressed as mean ± SD, or median [interquartile range], and categorical variables as numbers (percentages). Comparisons between groups were analyzed by performing Student's *t* test, whereas a χ^2^ test was used for categorical variables.

ALT = alanine aminotransferase; AST = aspartate aminotransferase; CRP = C‐reactive protein; FRAX = fracture risk assessment tool; HOMA‐β = homeostasis model assessment of β‐cell function; K‐GDS = Korean geriatric depression score tool; K‐MMSE = Korean mini‐mental status examination; TBS = trabecular bone score; TSH = thyroid‐stimulating hormone (thyrotropin).

During follow‐up, fragility fractures occurred in 537 (25.6%) of the participants. There were 223 clinical fractures cases reported by patients, and 314 cases detected by X‐ray readings. Of 223 clinical fractures, 54 cases were vertebral fractures (2.4%), 77 were hip fractures (3.5%), and 92 were upper extremity fractures (4.1%). In addition, 128 (5.7%) participants died during follow‐up.

### Top‐20 predictors by outcomes

The top‐20 predictors using the CatBoost model for each outcome ordered by feature importance are listed in Table 2. Total hip BMD was the most important predictor of fracture. Lumbar spine and femur neck BMD were important predictors of fracture along with total hip BMD. Surprisingly, a subjective arthralgia score, serum creatinine, and homocysteine levels were the next important predictors of fracture. Aspartate aminotransferase, lumbar TBS, fasting glucose, age, TG levels, and the K‐MMSE score, reflecting cognitive function, were also high‐ranking predictors of fracture. Subsequently, C‐reactive protein (CRP), BMI, age of menarche, platelet count, income status, history of previous fracture, thyroid‐stimulating hormone (TSH) level, and K‐GDS scores were determined in the machine‐learning algorithms to be high‐ranking predictors.

**Table 2:** Top‐20 Features Derived From the CatBoost Model

Ranking	Risk factor	Feature importance
1	Total hip BMD	0.222
2	Lumbar spine BMD	0.112
3	Femur neck BMD	0.101
4	Arthralgia score	0.100
5	Creatinine	0.090
6	Homocysteine	0.086
7	AST	0.076
8	Lumbar spine TBS	0.072
9	Fasting glucose	0.068
10	Age	0.062
11	Triglyceride	0.062
12	K‐MMSE	0.061
13	CRP	0.060
14	BMI	0.058
15	Menarche	0.055
16	Platelet	0.049
17	Income status	0.043
18	Previous fracture history	0.041
19	TSH	0.040
20	K‐GDS	0.038

AST = Aspartate aminotransferase; CRP = C‐reactive protein; K‐GDS = Korean geriatric depression score; K‐MMSE = Korean mini‐mental status examination; TBS = trabecular bone score; TSH = thyroid‐stimulating hormone (thyrotropin).

**Figure 2:**
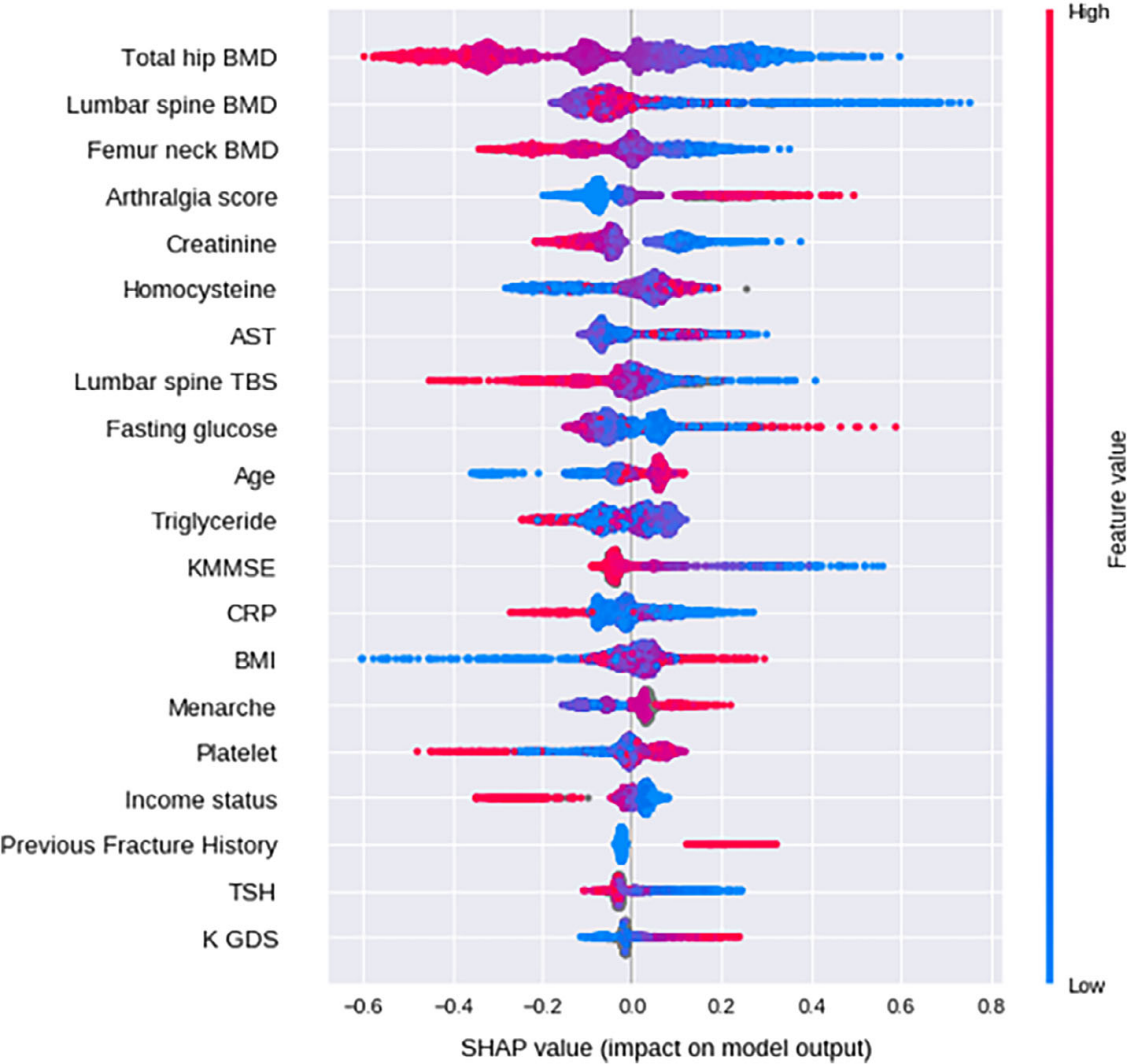
Impact of features on prediction model output. Red and blue colors represent high and low levels of each predictor. The *x*‐axis represents the SHAP value. A positive SHAP value means likely to have a fracture; a negative value means unlikely to have a fracture. AST = aspartate aminotransferase; TSH = thyroid‐stimulating hormone (thyrotropin); TBS = trabecular bone score; KMMSE = Korean mini‐mental status examination; CRP = C‐reactive protein; K‐GDS = Korean geriatric depression score; SHAP = Shapley additive explanations.

**Figure 3:**
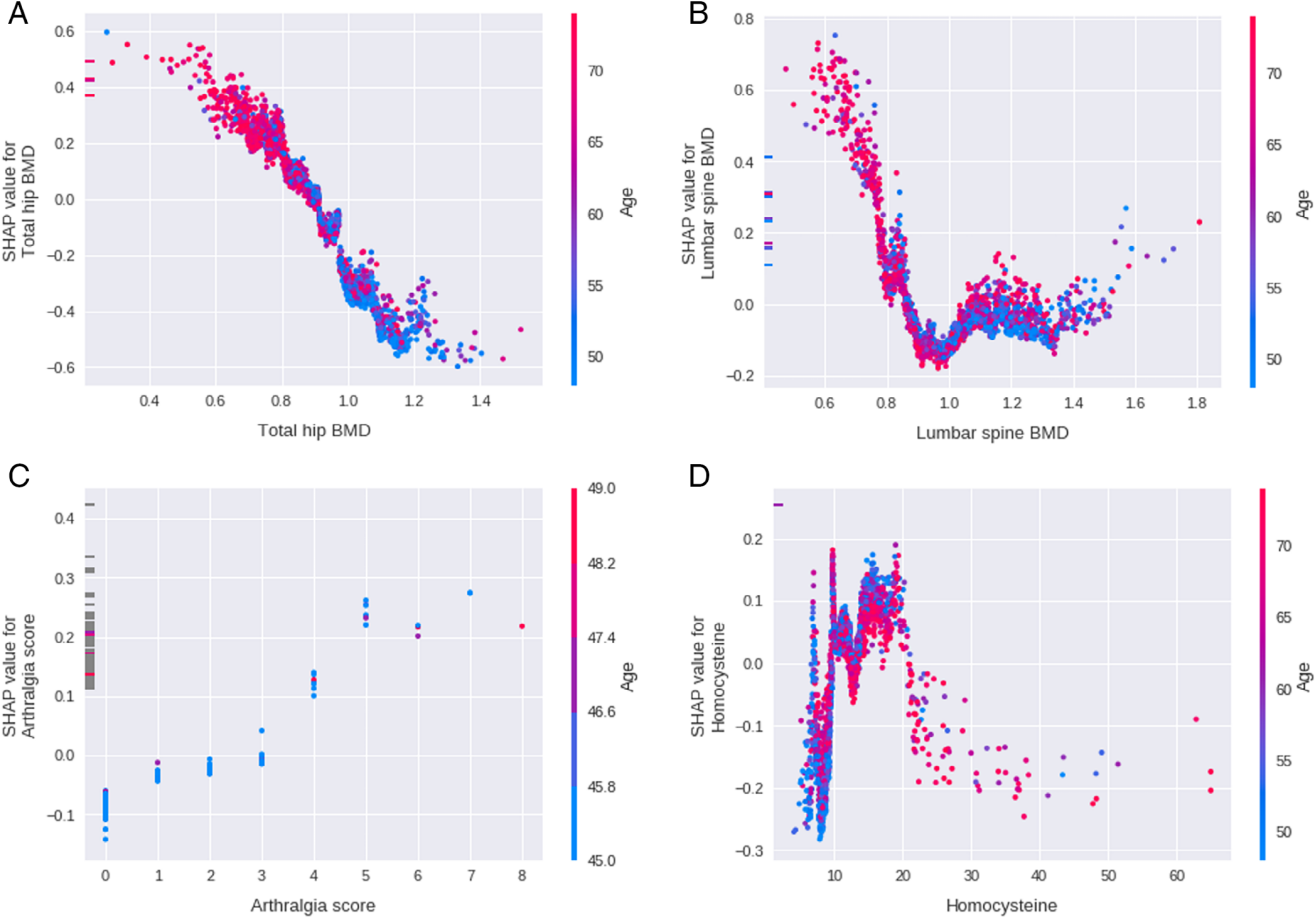
Impact on prediction model output of (*A*) total hip BMD, (*B*) lumbar spine BMD, (*C*) subjective arthralgia score, and (*D*) homocysteine level. Red and blue colors represent old and young age. The *y*‐axis represents the SHAP value. A positive SHAP value means likely to have a fracture; a negative value means unlikely to have a fracture. SHAP = Shapley additive explanations.

As illustrated in Fig. 2, decreased BMD and TBS were related to an increased fracture risk with a large impact on the model. Also, increased arthralgia score, low level of serum creatinine, mild elevation of homocysteine, high fasting glucose, and age were related to an increased fracture risk. Decreased cognitive function, income status, TSH, increased CRP, BMI, and age of menarche contributed to an increased fracture risk. The phenomenon was supported in partial dependence plots as given in Fig. 3, which demonstrate decreasing total hip BMD; lumbar spine TBS had an increasing model contribution value to the fracture prediction (Fig. 3 *A*,*B*). As can be seen in Fig. 3 *A* and *B*, patients of younger age tended to have higher BMD and lower TBS. In Fig. 3 *C* and *D*, partial dependence plots show that increased arthralgia score and mildly increased homocysteine level had an increased model contribution value to fracture prediction.

### Performance of the model

Compared with conventional models such as logistic regression and SVM, the CatBoost machine‐learning model had the best performance in predicting fractures (Table 3). The AUC of the CatBoost model was significantly higher than those of the logistic regression model and the SVM model in total fracture prediction, vertebral fracture prediction, and hip fracture prediction (*p* < 0.001 for all).

**Table 3:** Performance in AUC of Machine‐Learning Models

	Total fracture	Vertebral fracture	Hip fracture
CatBoost model with all variables	0.688^b^^,^ c (0.687–0.688)	0.684^b^^,^ c (0.683–0.684)	0.656^b^^,^ c (0.655–0.656)
CatBoost model with top‐20 variables	0.688^d^^,^ e (0.687–0.688)	0.656^a^^,^ d ^,^ e (0.655–0.656)	0.653^a^^,^ d ^,^ e (0.653–0.653)
Logistic regression model with all variables	0.614 (0.612–0.616)	0.663 (0.661–0.664)	0.606 (0.598–0.614)
Logistic regression model with top‐20 variables	0.565^a^ (0.562–0.567)	0.628^a^ (0.627–0.630)	0.622^a^ (0.615–0.630)
SVM model with all variables	0.500 (0.500–0.501)	0.502 (0.501–0.502)	0.502 (0.502–0.502)
SVM model with top‐20 variables	0.542^a^ (0.540–0.544)	0.563^a^ (0.561–0.565)	0.503 (0.497–0.510)

Evaluation of the performance of the prediction models were done in area under the curve (AUC) score with randomly selected threefold cross‐validation for 1000 times.

SVM = support vector machine.

a Refers to *p* < 0.001 of model with top‐20 variables compared with model with all variables.

b Refers to *p* < 0.001 of CatBoost model compared with the logistic regression model.

c Refers to *p* < 0.001 of CatBoost model compared with the SVM model.

d Refers to *p* < 0.001 of CatBoost model with top‐20 variables compared with the logistic regression model with top‐20 variables.

e Refers to *p* < 0.001 of CatBoost model with top‐20 variables compared with the SVM model with the top‐20 variables.

The CatBoost model with the top‐20 variables showed a similar performance in total fracture prediction and a more reduced performance in vertebral and hip fracture prediction than the model with all variables. Logistic regression with the top‐20 variables showed more reduced performance in total and vertebral fracture prediction, but better performance in hip fracture prediction than the model with all variables. The SVM model with the top‐20 variables showed better performances in total fracture and vertebral fracture prediction than the model with all variables. Among the three models with the top‐20 variables, the CatBoost model with the top‐20 variables showed the best performance with an AUC of 0.688 compared with the logistic regression model (AUC of 0.565) or the SVM model (AUC of 0.542) with the top‐20 variables (Table 3).

As the CatBoost model had the best performance, the performance of the CatBoost model was compared with the FRAX score model (Table 4). For the total fracture category, the AUC of the CatBoost machine‐learning model was 0.687, which was significantly better than the FRAX score with TBS data (0.663, *p* < 0.001). For the hip fracture category, the AUC of the CatBoost model was 0.656, which was also significantly higher than the FRAX score with TBS data (0.549, *p* < 0.001). Comparing the SVM and logistic regression model with FRAX (major fracture with TBS, AUC 0.663), both the logistic regression model (AUC 0.614) and SVM model (AUC 0.500) had significantly lower AUC values (*p* < 0.001 for both; Tables 3 and 4).

**Table 4:** Performance in AUC of Machine Learning and FRAX Score

	Total fracture	*p* a	Hip fracture	*p* a
Machine‐learning model (CatBoost)	0.688 (0.687–0.688)		0.656 (0.655–0.656)	
FRAX (major, without BMD), %	0.638	<0.001	‐	‐
FRAX (major, with BMD), %	0.660	<0.001	‐	‐
FRAX (major, with TBS), %	0.663	<0.001	‐	‐
FRAX (hip, without BMD), %	‐	‐	0.528	<0.001
FRAX (hip, with BMD), %	‐	‐	0.545	<0.001
FRAX (hip, with TBS), %	‐	‐	0.549	<0.001

AUC = area under the curve; FRAX = fracture risk assessment tool; TBS = trabecular bone score.

a FRAX scores compared with the machine‐learning model.

## Discussion

This is the first study to develop and evaluate a fracture prediction model with the CatBoost machine‐learning method in a longitudinal community‐based cohort study. The prediction model suggested the top‐20 risk factors of the fracture including well‐known factors such as total hip, lumbar, and femur neck BMD; TBS; body weight; age of menarche; age; and history of previous fractures, as well as lesser‐known novel factors such as arthralgia subjective score, homocysteine, CRP, TG levels, K‐GDS score, homeostasis model assessment of β‐cell function (HOMA‐β), and income status. The performance of the CatBoost model was better in predicting total fracture and hip fracture than the FRAX score, and better than conventional models such as logistic regression and the SVM model. Also, the CatBoost model constructed with only the top‐20 variables showed similar performance as the model with all variables.

Our study has clinical importance in developing a fracture prediction model with machine learning in a large‐sized longitudinal cohort. There are few machine‐learning studies in predicting osteoporotic fracture.^7^, ^9^, ^24^ In one study, which includes QCT and BMD data, a gradient boosting machine‐learning model was developed to predict fracture in 332 participants. The performance of the study improved significantly after applying a gradient boosting machine method (AUC of each variable: 0.61, AUC of gradient boosting model: 0.81).^7^ Although the study had a small number of patients, a strength of the study is that it includes bone BMD and QCT data to improve the model performance with sufficient follow‐up duration. However, as the top risk features were well‐known variables such as BMD, the study could not suggest novel clinical features from the model. Also, the study did not compare the AUC with a conventional risk prediction model such as FRAX. Recently, another study reported a machine‐learning model that predicts quantitative ultrasound speed of sound using genome‐wide association data.^9^ However, the model has limitations in predicting fracture without BMD. This study could be clinically meaningful in that it is the first study to develop a machine‐learning model for predicting fracture using a large‐sized prospective cohort with BMD and TBS data.

The CatBoost model was used as a machine‐learning technique in this study. The CatBoost model is a modification of a gradient boosting method, a machine‐learning technique that provides superb performance in many tasks. CatBoost, as the name suggests, entails statistical techniques to learn categorical features, which have substantially different characteristics to numerical features. Furthermore, it prevents overfitting by using unbiased estimates for the gradients.^10^ The CatBoost algorithm was chosen as the data set comprises many categorical variables (eg, sex, smoking status, income level), and to ensure the generalizability of the model by minimizing overfitting.

Notably, the study suggested novel high‐ranked factors in fracture prediction. First, the subjective arthralgia score was ranked as the fourth most essential feature in the fracture prediction model, which was higher than the lumbar TBS score. Also, patients who have had fractures showed a significantly higher subjective arthralgia score than those who did not. The associations of arthralgia with fracture have not been well‐studied, but there has been speculation that there are links between pain neuropeptides and the pathological process of osteoporosis and bone remodeling.^25^ A recent study reported that participants with chronic arthralgia were likely to be diagnosed with spinal osteoporosis with a relative risk ratio of 4.12.^26^ Previous studies have shown that the treatment of osteoporosis alleviated arthralgia in patients with osteoporosis, as well as reduced bone resorption and improved BMD.^27^, ^28^, ^29^ This is hard to validate in this study because our cohort did not include bone turnover markers; further investigation is needed to clarify the issue. It could also be possible that the participants with arthralgia are more likely to fall because of the pain itself.^30^ As expected, participants with osteoarthritis complained of more severe arthralgia than those without osteoarthritis (arthralgia score 2.92 ± 2.76 in patients with arthritis [*n* = 173], 1.43 ± 3.05 in patients without arthritis, *p* < 0.001). The osteoarthritis may also predispose the sarcopenia and risk of falls,^31^ whereas it may not be in the top variables for predicting fracture because of the collinearity with the arthralgia score. The degree of the arthralgia may have a predictive value for fracture as well for this study, and it could be used as an early marker for increased bone resorption and fractures.

Hyperhomocysteinemia was also a highly ranked predictive factor for osteoporosis in this study. Mildly elevated plasma level of homocysteine is a common condition, and it is reported to be associated with an increased risk of fractures.^32^, ^33^ In this study, it is notable that homocysteine is highly ranked, higher than conventional risk factors such as age, body weight, and lumbar TBS score. Homocysteinemia is known to be related to the disturbance of collagen linking of the bone by reacting with aldehyde to form a stable thiazide ring in the collagen‐linking process.^34^ In patients with homocystinuria, which implicates a high circulating level of homocysteine, a lower amount of collagen‐linking was found than in normal participants.^35^ However, it was also reported that a low estradiol level was associated with high homocysteine levels.^36^ Also, low serum creatinine as a high ranked predictive factor implies that serum creatinine could be used as an indicator of low muscle mass to predict fracture. As serum creatinine more strongly correlates with lean mass than with total body weight,^37^ low serum creatinine in elderly patients could represent low muscle mass. It could be an easily accessible method in clinical practice to reflect muscle mass, especially in an older population.

In this study, the machine‐learning model showed a similar or better performance than the FRAX method for fracture prediction. FRAX is a widely accepted, excellent tool not just to calculate the 10‐year risk of fracture, but it also includes parameters that can be reversed with treatment. Therefore, improving the model with a machine‐learning method is clinically meaningful. The performance of the FRAX model in the study was similar to previous reports.^38^, ^39^ Nevertheless, the performance of the machine‐learning model, especially in predicting hip fracture, was significantly better than that of FRAX, but not in predicting vertebral fractures. It could be because the onset of a hip fracture is relatively accurate, whereas the onset of a vertebral fracture is less accurate because of the nature of the fracture. Although we tried to overcome this limitation by finding vertebral fractures in X‐rays, there is the possibility that the onset time of the vertebral fracture might not be punctual. Therefore, the model is more suitable for the prediction of total fractures or hip fractures in particular than for the prediction of vertebral fractures. Also, FRAX may not be a fair model for the comparison because of the various follow‐up periods in the study, considering that the FRAX was initially designed to predict a 10‐year risk. Therefore, the interpretation of the performances could be somewhat different as the FRAX might be underestimated because of the design of the cohort, but still have excellent performance. It could imply that the machine‐learning model may have its main strength in finding novel prediction markers with acceptable performance.

Furthermore, models with the top‐20 variables showed a noninferior performance. This result could be because the few main variables led to the performance of the model, whereas the remaining variables did not have substantial roles because of the collinearity. The phenomenon can also be seen in other studies. In one recent study predicting cardiovascular events using machine learning, a model with top‐20 variables was also used and showed excellent performance compared with a model with all variables.^5^ In addition, as the top variables mostly contributed to the model, it was shown that a model with only nine variables (forwardly selected) had better performance than a model with all variables. Besides, the model with few variables makes it more practical in the clinical field to validate in other cohorts.

This study has some strengths. First, this is the first study to evaluate a fracture prediction model using machine learning in a large prospective cohort, including BMD. The cohort has its strengths in that the population of the cohort is homogenous, prospectively followed‐up with BMD, TBS, and thorough anthropometric measures. Also, the model suggested novel high‐ranking factors in fracture prediction, which could be considered in clinical research and practice. Developing and validating a simplified model with the top‐20 factors is also a strength of this study, which makes the model practical and suggests the possibility of use in clinical practice.

The study has some limitations. The study suggested novel predictors included in the top‐20 models, which are not common measurements in standard clinical practice. Therefore, it may not be easy to apply the model in a real‐world setting. Also, the study lacks the data of bone turnover markers and hormone data such as estrogen and testosterone. Because these data are now being measured in a single cohort, future studies could be improved by including bone turnover markers and hormone data. Also, because of the inclusion of morphometric fracture events, old baseline age of this study, and the characteristics of the rural farmland community, the incidence of a fragility fracture was higher than the national medical claim data in Korean people older than 50.^40^, ^41^, ^42^ As the model was not based on a time‐dependent analysis, it is a limitation that the model could not suggest the predicted time to the fracture. Further studies with survival analysis will be needed. In addition, the study was analyzed in a homogenous group, which requires further validation in other ethnicities.

This study is the first study of a fracture prediction model with the CatBoost machine‐learning method in a longitudinal community‐based cohort. In predicting total fractures and hip fractures, the performance of the CatBoost model was better than using the FRAX score. The prediction model suggested novel predictors such as an arthralgia subjective score and homocysteine levels with conventional predictors in fracture prediction. Therefore, this study is clinically meaningful in suggesting a model with acceptable performance and in proposing a ranking of predictors with a novel methodology. Further validation studies in various groups and large cohorts are needed to improve the model.

## Disclosures

All authors state that they have no conflicts of interest.
